# CLTNet: A Hybrid Deep Learning Model for Motor Imagery Classification

**DOI:** 10.3390/brainsci15020124

**Published:** 2025-01-27

**Authors:** He Gu, Tingwei Chen, Xiao Ma, Mengyuan Zhang, Yan Sun, Jian Zhao

**Affiliations:** 1 College of Computer Science and Technology, Changchun University, Changchun 130022, China; guh@ccu.edu.cn (H.G.); chentingwei2000@gmail.com (T.C.); maxiao19990302@gmail.com (X.M.); zmy901@outlook.com (M.Z.); sunyan.ee@outlook.com (Y.S.); 2 Key Laboratory of Intelligent Rehabilitation and Barrier-Free Access for the Disabled, Ministry of Education, Changchun 130022, China; 3 Jilin Provincial Key Laboratory of Human Health State Identification and Function Enhancement, Changchun 130022, China; 4 Jilin Rehabilitation Equipment and Technology Engineering Research Center for the Disabled, Changchun 130022, China

**Keywords:** brain–computer interface, motor imagery, deep learning, convolutional neural network, long short-term memory network, multi-head attention

## Abstract

Background: Brain–computer interface (BCI) technology opens up new avenues for human–machine interaction and rehabilitation by connecting the brain to machines. Electroencephalography (EEG)-based motor imagery (MI) classification is a key component of BCI technology, which is capable of translating neural activity in the brain into commands for controlling external devices. Despite the great potential of BCI technology, the challenges of extracting and decoding brain signals limit its wide application. Methods: To address this challenge, this study proposes a novel hybrid deep learning model, CLTNet, which focuses on solving the feature extraction problem to improve the classification of MI-EEG signals. In the preliminary feature extraction stage, CLTNet uses a convolutional neural network (CNN) to extract time series, channel, and spatial features of EEG signals to obtain important local information. In the deep feature extraction stage, the model combines the long short-term memory (LSTM) network and the Transformer module to capture time-series data and global dependencies in the EEG. The LSTM explains the dynamics of the brain activity, while the Transformer’s self-attention mechanism reveals the global features of the time series. Ultimately, the CLTNet model classifies motor imagery EEG signals through a fully connected layer. Results: The model achieved an average accuracy of 83.02% and a Kappa value of 0.77 on the BCI IV 2a dataset, and 87.11% and a Kappa value of 0.74 on the BCI IV 2b dataset, both of which outperformed the traditional methods. Conclusions: The innovation of the CLTNet model is that it integrates multiple network architectures, which offers a more comprehensive understanding of the characteristics of the EEG signals during motor imagery, providing a more comprehensive perspective and establishing a new benchmark for future research in this area.

## 1. Introduction

Brain–computer interface (BCI) is a promising technology that enables two-way communication of information through neural signals linking brain activity with external devices (e.g., drones or prosthetics) [1]. The core of BCI technology lies in the ability to recognize the different activity states of the brain and translate them into specific control signals or commands, enabling people to bypass the traditional muscle control pathway and directly use their mind to interact with the external world [1]. Electroencephalography (EEG) is widely used in BCI systems as a non-invasive, low-cost, portable technology with high temporal resolution. Nowadays, it has become increasingly common to use these systems to control robotic devices to perform complex tasks that can be useful in daily life [2]. EEG is able to record the electrical signals of the brain in different states in real time, including the responses of event-related potentials (ERPs) [3], steady-state visual evoked potentials (SSVEPs) [4], and sensorimotor rhythms (SMRs) [5]. By analyzing EEG signals, researchers have been able to more accurately identify patterns of brain activity associated with actual movement, or motor imagery (MI), which serves as a mental rehearsal of motor behavior without the need to actually perform the physical movement. The SMRs generated by the human brain while performing MI tasks exhibit the phenomenon of event-related desynchronization/resynchronization (ERD/ERS), which is characterized by an increase in ipsilateral energy and a decrease in contralateral energy, and has received particular attention. Since MI is organized according to the topology of motor neurons, the ERD/ERS phenomenon involves multiple discharge signals from motion-sensing neurons. Therefore, decoding EEG signals recorded during MI has become a critical and challenging problem for MI-BCI [6].

Moreover, MI-EEG-based decoding is not only widely used in medical and rehabilitation fields [7], but has also emerged as a promising technology in non-medical fields such as virtual reality [8], gaming [9], and robotic arm control [10]. Despite the progress of deep learning (DL) in improving classification accuracy, the low signal-to-noise ratio, susceptibility to noise interference, and large individual differences of EEG signals still exist [11]. Therefore, feature extraction for motion imagery and improving the accuracy and robustness of BCI systems in MI classification are still key issues to be addressed in future research.

Meanwhile, a variety of algorithms for MI classification have been developed in the field of machine learning (ML) techniques. The ML techniques focus on three key phases: preprocessing, feature extraction, and classification of MI-EEG signal data. In the feature extraction phase, Common Spatial Pattern (CSP) methods [12] and Power Spectral Density (PSD) [13] features are widely developed and applied. In the classification phase, the features extracted from the EEG trials are fed into different classifiers, which include Support Vector Machines (SVMs) [14], Decision Trees [15] and Linear Discriminant Analysis (LDA) [16] to classify the signals into different MI tasks. Despite the success of these methods in MI classification, their performance relies heavily on hand-crafted feature extraction processes. These handcrafted features may not only be unstable, but also lead to unsatisfactory classification results in some cases. Therefore, the development of more stable and automated feature extraction methods, as well as the exploration of more advanced classification techniques, has become a key research direction to improve the performance of MI-EEG signal classification.

DL has shown great advantages in various fields, such as computer vision [17], natural language processing [18], FinTech [19], and autonomous driving analytics [20]. When dealing with the complex problem of MI-related EEG task classification, researchers want to use deep learning methods to automatically learn complex features from data, reduce the instability of manual feature extraction, and achieve stronger generalization ability. Therefore, researchers have shifted from traditional ML methods to DL techniques to decode MI-EEG signals more efficiently [21]. Meanwhile, researchers have also proposed various hybrid methods to overcome the limitations of MI-EEG decoding by combining the respective advantages of different DL methods [22,23,24] to improve the accuracy and robustness of MI-EEG signal classification.

In recent years, hybrid DL models incorporating convolutional neural networks (CNNs) have made significant progress in the field of BCI for MI tasks [21]. CNNs efficiently capture local features of the data through mechanisms such as local sensory fields, weight sharing, and pooling to achieve efficient classification. Researchers typically use CNN in combination with other deep learning models to improve performance. For example, the BrainGridNet framework, a two-branch deep convolutional neural network proposed by Wang et al. [25] performs well in both time and frequency domain analysis, especially in the frequency domain, with an accuracy of 80.26% and a kappa value of 0.753. However, there is still room for improvement in the framework’s ability to capture long-term dependencies. Arı et al. [26] developed an NF-EEG model that can directly process raw EEG signals without preprocessing to achieve signal classification for multiple classes of motor imagery. Liang et al. [27] proposed an EEG-CDILNet model that incorporates deeply separable convolution and CDIL techniques to successfully extract EEG signal features and reduce the number of model parameters, but only achieved less than 80% classification accuracy in a four-classification task on the BCI IV 2a dataset. Dai et al. [28] proposed a mixed scale CNN architecture including data augmentation to improve the classification performance, but the unstable performance on different datasets limits its wide applicability in practical applications. Hou et al. [29] developed a new method combining ESI techniques and CNNs, which significantly improves the classification accuracy of EEG motor imagery task, but requires significant training time. These studies show that although a CNN performs well in local feature extraction, it still suffers from the shortcomings of limited sensory field and restricted feature expression, and therefore it can be improved by combining multiple feature extraction.

In contrast, the combination of recurrent neural networks (RNNs), especially long short-term memory (LSTM) networks, which can efficiently process time-series data, is widely used to extract temporal features for MI-EEG tasks. Amin et al. [30] proposed a model of attention mechanism combining lightweight CNNs and LSTM, which not only achieves a short computation time, but also achieves excellent accuracy on public competition datasets, with excellent accuracy. Khademi et al. [22] developed three hybrid models of CNNs and LSTM, which improved MI classification accuracy by combining the local recognition ability of CNNs and the recognition of complex temporal dependencies by LSTM. Li et al. [31] proposed a feature fusion algorithm combining CNNs and LSTM, which achieves parallel extraction of spatial and temporal features and implements the fusion at the fully connected layer for MI classification. Karimian et al. [32] introduced a TD-LSTM framework that fully utilizes LSTM and temporally distributed methods to extract spatiotemporal dependencies in EEG signals. Xu et al. [33] et al. proposed an LSTM-based recurrent neural network model for decoding multi-channel EEGs or ECoGs, which achieved the effective extraction of robust spatiotemporal features and significantly improved the performance of MI brain–computer interface systems. Although LSTM is capable of capturing long-term dependencies in EEG signals, it suffers from the shortcomings of being difficult to parallelize and requiring a large amount of data for training.

Although hybrid models combining CNNs and LSTM have made progress in improving the classification accuracy of EEG signals, these models can still be improved in terms of parallel training and capturing long-term dependencies in EEG signals. Transformer models have become an effective tool for researchers to handle complex patterns in EEG signals due to their ability to accurately identify key elements, process long-range dependencies, and parallelly handle a large number of data points. For example, Altaheri et al. [34] proposed the ATCNet framework utilizing multi-head self-attention, which not only extracts high-level temporal features of MI-EEG but also significantly enhances data processing effects. Liu et al. [35] proposed an end-to-end multi-scale visual Transformer neural network model MSVTNet, which first extracts multi-scale spatiotemporal features through the CNN model and then uses the Transformer architecture to capture global temporal correlations and cross-scale interaction information. Zhang et al. [36] proposed a MI-CAT model architecture that innovatively combines the self-attention and cross-attention mechanisms of the Transformer model to solve the problem of different domain distribution differences. Ma et al. [37] proposed a hybrid model combining CNN and Transformer architectures, where the CNN architecture is responsible for extracting local features and Transformer is responsible for perceiving global dependencies. Hu et al. [38] proposed a method combining a multi-scale feature extraction (MSFE) module and an adaptive temporal transformer (ATT) module, achieving the extraction of highly discriminative features across multiple bands and adaptively extracting the temporal dependencies of EEG signals. These research advances demonstrate the potential of Transformer models and their variants in the field of EEG signal decoding, but the time-varying nature and low signal-to-noise ratio characteristics of EEG signals may affect the performance of the models.

**Figure 1:**
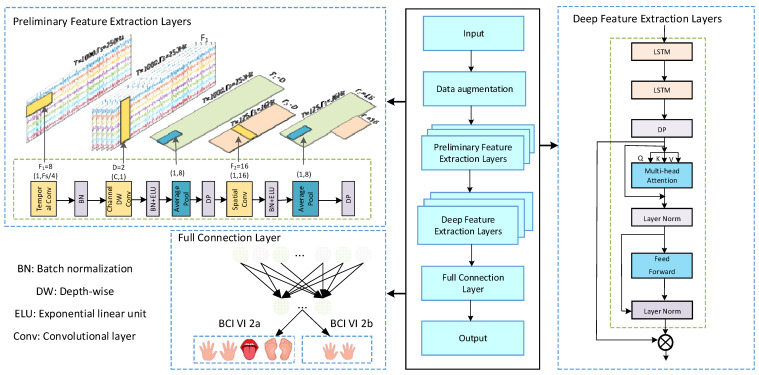
Deep learning architecture for hybrid network models of the CLTNet.

It can be seen that CNNs excel in feature extraction, and when processing EEG signals, CNNs can extract both temporal and spatial features of EEG data [39]. Moreover, through a multi-layer convolutional structure, CNNs are able to capture complex frequency domain characteristics, further enhancing their feature extraction capabilities for EEG signals [40]. LSTM is suitable for handling dynamic temporal information, and its unique gating mechanism allows it to capture dependency relationships in EEG signals over a longer time span [33]. The Transformer model is adept at modeling global dependencies through its multi-head attention mechanism [34,35,36]. In EEG analysis, transformers can more effectively capture global connections between time, space, and channels, and especially leverage their parallel computing advantages when dealing with large amounts of data to improve classification performance. Since CNNs, LSTM, and Transformers each have their own characteristics and strengths, combining these DL techniques is key to unleashing their potential and improving the model’s robustness and generalizability in the face of changes in the distribution of motor image categories and noise. Therefore, this paper proposes a hybrid model for deep learning with CLTNet, which covers the whole process from initial feature extraction to deep feature extraction, as shown in Figure 1.

In the preliminary feature extraction layer, temporal, spatial, and channel features are first efficiently extracted using a 1D CNN; in the deep feature extraction layer, the temporal features of EEG signals are accurately extracted using the modelling ability of the LSTM network on sequences, and the ability to comprehensively learn global features from EEG experiments is further enhanced using the transformer encoder. Finally, a quadruple classification of motor imagery EEG signals is performed in conjunction with the full connectivity layer. The main contributions of this work are as follows:

1. Proposed CLTNet model: a novel deep learning model, CLTNet, is designed by integrating CNN, LSTM, and Transformer modules to comprehensively extract local features, time-series features and global dependencies of motor imagery EEG signals.
2. Multi-dimensional feature extraction: in the preliminary feature extraction stage, CLTNet uses a CNN to extract local spatial, temporal, and channel features from EEG signals. In the deep feature extraction stage, the innovative fusion of LSTM and Transformer modules captures the dynamic changes and global characteristics of motor imagery EEG signals, respectively.
3. Validation of classification performance: experimental validation on publicly available EEG datasets demonstrates the stability and applicability of the CLTNet model for multitask classification, with strong generalization capabilities.
4. For the sake of reproducibility and further research, the code and trained models have been released at: https://github.com/ctwei-wed/CLTNet (accessed on 15 January 2025).

## 2. Materials and Methods

### 2.1. Dataset

**Figure 2:**
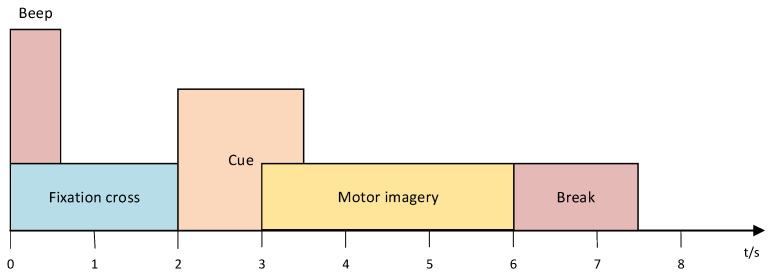
Processes within the motor imagery paradigm (example: BCI IV 2a).

In order to assess the validity and feasibility of the proposed CLTNet methodology, a comprehensive evaluation was conducted in this study by selecting two widely recognized benchmark MI BCI datasets: BCI Competition IV 2a (BCI IV 2a) [https://www.bbci.de/competition/download/competition_iv/BCICIV_2a_gdf.zip (accessed on 15 January 2025)] and BCI Competition IV 2b (BCI IV 2b) [https://www.bbci.de/competition/download/competition_iv/BCICIV_2b_gdf.zip (accessed on 15 January 2025)]. A comprehensive assessment was conducted. These datasets were chosen because they are widely cited in the literature and have similar MI experimental paradigms, which helps ensure the consistency and comparability of the assessment results, as shown in Figure 2.

Each session consisted of six runs, with 48 trials per run. Subjects imagined four categories of left-hand movements, right-hand movements, foot movements, and tongue movements in front of a computer screen, based on the direction of an arrow that appeared for approximately 1.25 s. Each trial lasted approximately 6 s from the appearance of a fixed cross to its disappearance, and with breaks, the average time to complete a trial was approximately 8 s. The results were summarized as follows.

(1) BCI IV 2a dataset

This dataset covers four different MI tasks: imagining movements of the left hand, the right hand, both feet, and the tongue. The dataset consists of 2 EEG experimental sessions involving 9 subjects, 1 EEG experimental session containing an array of 9 sample units, the first three units containing the EOG test data, and the 4th to 9th units containing the EEG data used for EEG analysis. These data were acquired through 22 Ag/AgCl electrodes spaced 3.5 cm apart, the signal was sampled at 250 Hz, and the data were band-pass filtered from 0.5 to 100 Hz with an additional 50 Hz trap filter activated to suppress line noise. The data acquisition was divided into two phases; the data from the first phase was used for model training, while the data from the second phase was used for model evaluation. Each acquisition phase contained 288 trials, 72 trials for each MI task.

(2) BCI IV 2b dataset

This dataset covers two different MI tasks: imagining left-handed and right-handed movements. The dataset consists of five EEG experimental sessions involving nine subjects, with one EEG experimental session containing an array of two to three EEG data sample units. Among them, the first two sessions were conducted in the no-feedback condition, and the last three sessions were conducted in the feedback condition. In each session, subjects performed the imagery task 60 separate times, for a total of 120 trials. The duration of each motor imagery task was 4 s. Three C3, Cz, and C4 bipolar EEG signals were acquired at a sampling frequency of 250 Hz in the range of 0.5 to 100 Hz for band-pass filtering, and a 50 Hz trap filter was set up at the time of recording using signal acquisition hardware.

#### 2.1.1. Data Pre-Processing

To improve the quality of the EEG signal data and optimize the model inputs for the BCI IV-2a and IV-2b datasets, a Z-score normalization preprocessing was performed using Matlab R2019a to ensure the consistency of the data scales and improve the efficiency of model training. First, the motion imagery samples in the dataset were selected; second, the data of each sample were stored in a three-dimensional (number of experiments, number of channels, number of sampling points) matrix; then, the EEG signals were extracted from the time range of 501~1500 ms, and only the critical EEG channels were retained. Finally, all sample data were merged to adjust the dimensionality and form a standardized data structure to provide high-quality data input for model training or data analysis. For the BCI IV 2a dataset, a data structure with dimensions (288, 22, 1000) was created, where 288 is the total number of trials, 22 is the number of channels, and 1000 is the number of sample points. For the BCI IV 2b dataset, the training and test sets formed a data structure with dimensions of approximately (400, 3, 1000) or (320, 3, 1000), respectively, where 400 and 300 are the total number of trials, 3 is the number of channels, and 1000 is the number of time points. These preprocessing steps provide standardized and quality data input to the classification model.

#### 2.1.2. Data Enhancement

**Figure 3:**
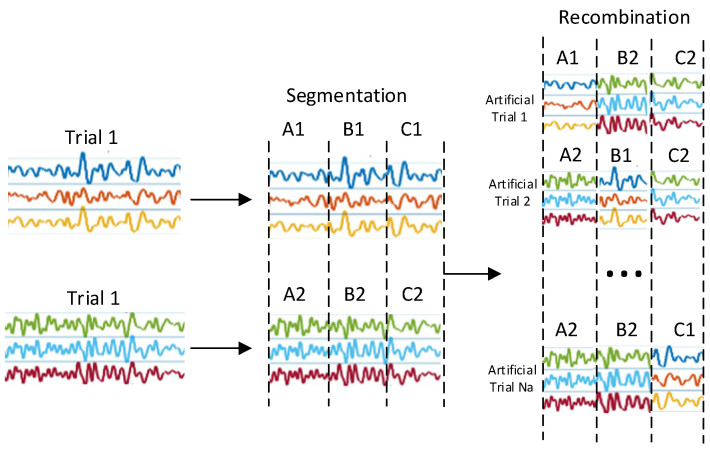
Data enhancement principle.

In the task of MI classification of EEG signals, this study addresses the problem of model overfitting caused by the small size of the dataset by adopting the time-domain segmentation and recombination (S&R) data enhancement method. It aims to expand the size of the dataset by generating new samples to enhance the recombination ability of the model. The specific steps include, firstly, defining a training set consisting of M trials $\Omega \in \left\{X_{i}^{\prime }\right\}$, $i\in \left[1,M\right]$, where each trial $X_{i}^{\prime }\in R^{C\times T}$ denotes an EEG trial data, C is the number of signal channels, and T is the number of time steps. Then, each trial dataset is uniformly divided into K consecutive segments, each segment containing $\frac{T}{K}$ time steps. Finally, these segments are randomly selected from different trials of the same category and restructured in the original time order to generate a new artificial trial sample, as shown in Figure 3.

After time-domain segmentation and recombination, the resulting augmented data $X_{i}$ can be expressed as:

(1) $$X_{i}^{\sim }=\left[X_{1}^{R},X_{2}^{R},...,X_{K}^{R}\right]$$

where $R_{K}$ is an integer index of training trials randomly selected from the training set range [1, *M*], $X_{K}^{R}$ denotes the *K*th segment selected from the $R_{K}$ th trial, and the time length of the segment is $\frac{T}{K}$.

The labels ${\tilde{y}}_{i}$ of the generated augmented data are kept consistent with the original labels:

(2) $${\tilde{y}}_{i}=y_{i}$$

### 2.2. Preliminary Feature Extraction Layer

The design of the convolution module in this study is inspired by the architecture of EEGNet [41], but with improvements in key implementation aspects. A strategy of decomposing the 2D convolution operation into two 1D components, temporal and spatial convolution, was adopted, and deep convolution was introduced to increase the flexibility and effectiveness of feature extraction. In contrast to the separable convolution used in EEGNet, the 2D convolution operation chosen in this study demonstrates better performance. As shown in Figure 1, the CNN module also differs from EEGNet in its parameter configuration, and the classification performance of the model is further improved by adjusting key parameters.

First, the first layer performs temporal convolution through a filter of size (1, $K_{C1}$), where $K_{C1}$ = 16 is the length of the filter on the time axis, set to one-fourth of the experimental data sampling rate (64 for BCI IV 2a). The design takes into account the characteristics of the experimental data and the task requirements, so that the filters can effectively extract the temporal information related to frequencies above 4 Hz. After the processing of this layer, the output of $F_{1}$ is a temporal feature map. The second layer is a deep convolutional layer, using $F_{2}$ a filter of size (*C*,1) for channel feature extraction, where *C* = 22, set as the number of channels of the experimental data. The aim is to reduce the computational complexity while improving the sensitivity of the model to spatio-temporal features, which is suitable for dealing with complex spatio-temporal dependencies in EEG signals. Therefore, the output from the second layer is $F_{1}\times D$ feature maps, where *D* = 2 indicates that each temporal feature map is connected with two filters, corresponding to the temporal feature maps in the previous layer. The second layer outputs a lower sampling rate of 32 Hz through an average pooling layer of size (1, 8), which not only reduces the computational complexity and the number of parameters, but also preserves the key features. The third layer uses $F_{2}$ filters of size (1, $K_{C2}$), with $K_{C2}$ = 16, so that the sense field of the filter covers a time range of 500 ms in order to capture the key timing features associated with MI activity at a sampling rate of 32 Hz. Finally, an average pooling layer of size (1, $P_{2}$) is used to reduce the sampling rate to 32 Hz/$P_{2}$. $P_{2}$ regulates the length of the EEG feature sequence to provide input to the subsequent LSTM module.

The dropout operation is applied after the two average pooling layers, and the dropout probability is reduced to 0.3 to suppress overfitting while retaining enough neurons to participate in the training to achieve better generalization performance. Finally, the convolution module generates the feature mapping $S\in R^{T_{C}\times d}$, where $T_{C}$ is the length of the high-level feature representation for the EEG trials, with the following formula:

(3) $$T_{C}=\frac{T}{8\times P_{2}}$$

where *T* is the time sample of the original EEG trial 1125, *d* = 16 is the number of channels and provides rich EEG feature information for subsequent deep learning models.

### 2.3. Deep Feature Extraction Layer

#### 2.3.1. LSTM Module

**Figure 4:**
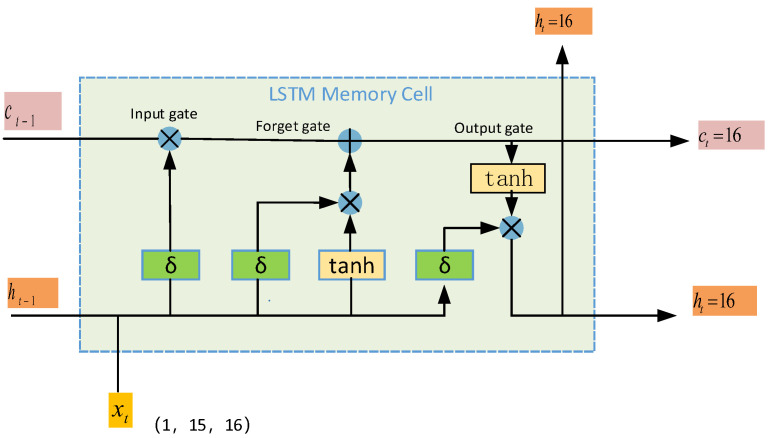
LSTM module structure.

LSTM is a special type of RNN that is best suited for analyzing temporal and sequential data [12]. To capture the time-dependent features of EEG signals, this study introduces a two-layer stacked LSTM after the CNN module. The LSTM, with its unique gating mechanism and cellular state design, has a significant advantage in dealing with sequential data, as shown in Figure 4.

In this case, input gates ($i_{t}$), forgetting gates ($f_{t}$), and output gates ($o_{t}$) are used to implement the control of state updates and information flow. The input gate determines the effect of the current input on the memory state, the forgetting gate selects the historical information to be discarded, and the output gate controls which information is output from the hidden state. At each time step, the LSTM unit dynamically adjusts the representation of the signal by updating the memory state ($c_{t}$) and the hidden state ($h_{t}$) to efficiently capture the dependencies in the time series. The formula is as follows:

(4) $$i_{t}=\sigma (W_{xi}x_{t}+W_{hi}h_{t-1}+b_{i})$$

(5) $$f_{i}=\sigma (W_{xf}x_{t}+W_{hf}h_{t-1}+b_{f})$$

(6) $$o_{t}=\sigma (W_{xo}x_{t}+W_{ho}h_{t-1}+b_{o})$$

(7) $$c_{t}=f_{t}.c_{t-1}+i_{t}.c_{t}$$

(8) $$h_{t}=o_{t}\cdot \tanh(c_{t})$$

Each gate of the LSTM uses a sigmoid activation function (σ) to calculate the corresponding gating value and adjusts the internal state based on information from the input sequence.

A two-layer stacked LSTM with 16 hidden units per LSTM layer is introduced in this study to efficiently model the time series of the input data. The features extracted by the CNN module with the shape of (16, 1, 15) are appropriately processed as inputs to the LSTM, which further models the temporal dimensions and generates feature representations that are consistent with the length of the input sequence. To reduce the risk of overfitting, a dropout layer is added after the LSTM output, and the dropout ratio is set to 0.5. This design enhances the model’s ability to handle complex temporal features, which is particularly suitable for modelling dynamic changes in EEG data.

#### 2.3.2. Transformer Module

The Transformer module is able to efficiently capture the dependencies between elements at different locations in the input sequence in the EEG signal classification task through its self-attention mechanism and multi-head attention mechanism (MHA), thus compensating for the shortcomings of preliminary feature extraction. In contrast to traditional sequential processing, the Transformer module constructs the interrelationships of elements within a sequence using the global self-attention mechanism, which makes it more advantageous in dealing with sequence data with long temporal dependencies and complex structures.

**Figure 5:**
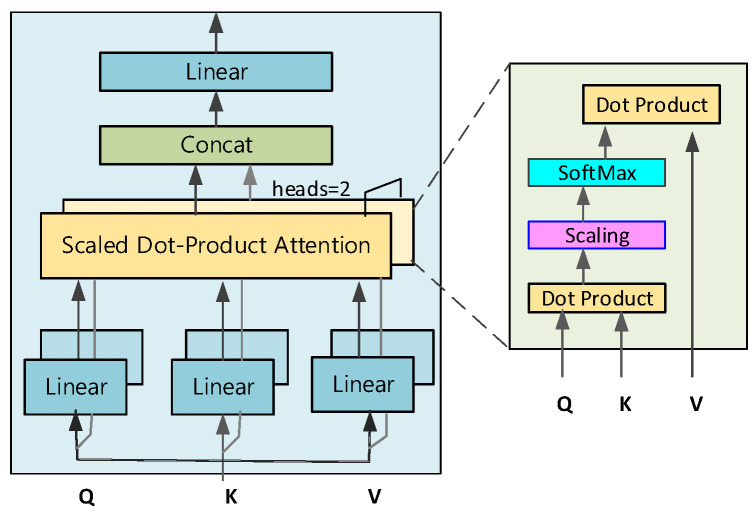
Transformer encoder architecture.

In this study, the encoder part of the Transformer module is used for EEG signal classification. The encoder consists of several stacked layers, each containing two main sub-modules: a multi-head self-attention mechanism and a feed-forward neural network. The overall architecture of the transformer encoder is shown in Figure 5.

The MHA mechanism is used to enhance the ability to capture the global time dependence of EEG signals, a property that effectively complements the convolutional modules in terms of sensory field. The MHA consists of multiple self-attentive heads, each of which transforms the input data by applying a unique weight matrix, which in turn produces three core components: query (*Q*), key (*K*), and value (*V*). The formula is as follows:

(9) $$Q_{i}=SW_{i}^{Q}\in R^{T_{C}\times d_{k}},W_{i}^{Q}\in R^{d\times d_{k}}$$

(10) $$K_{i}=SW_{i}^{K}\in R^{T_{C}\times d_{k}},W_{i}^{K}\in R^{d\times d_{k}}$$

(11) $$V_{i}=SW_{i}^{V}\in R^{T_{C}\times d_{k}},W_{i}^{V}\in R^{d\times d_{k}}$$

where $W_{i}^{Q}$, $W_{i}^{K}$ and $W_{i}^{V}$ are the projection matrices of the query, key, and value of the ith header, respectively. In calculating the attention weights, the query *Q* and the key *K* are subjected to dot product operation to get the similarity between them, and the dot product value is prevented from being too large by dividing it by the normalization factor $\sqrt{d_{k}}$. Next, the Softmax function is applied to normalize the normalized dot product to obtain the attentional weights for each key pair value $V_{i}$. Finally, these weights are used to weight and sum the values *V* to obtain the output of each attention header. $Z_{i}$ The formula is calculated as follows:

(12) $$Z_{i}=SA(Q_{i},K_{i},V_{i})=Soft\max\left(\frac{Q_{i}K_{i}^{T}}{\sqrt{d_{k}}}\right)V_{i}$$

The MHA mechanism allows the model to process information from different locations and representational subspaces in parallel. By performing multiple SA operations simultaneously, each focusing on a different aspect of the input data, it provides a comprehensive analysis of the input from multiple perspectives. The MHA then merges the outputs of these independent SA heads through a linear transformation to achieve information integration. In this way, the model is able to identify and capture dependencies in the data in multiple dimensions, improving its representational capabilities, especially when dealing with complex EEG time-series data, and better capturing global and local features. Its formula is as follows:

(13) $$MHA(Q,K,V)=Concat(Z_{1},Z_{2},...,Z_{h})W^{O}\in R^{T_{C}\times d_{k}},W^{O}\in R^{hd_{k}\times d}$$

where *h* is the number of attentional heads. With the introduction of the MHA mechanism, each node in the network has access to the global receptive field, allowing the model to extract and integrate information from the entire input sequence. After processing by the MHA module, the output features are merged with the original input features *S* by residual linking, followed by the application of layer normalization (LN) to normalize the individual features. The output of the MHA mechanism can be expressed as follows:

(14) O=LN(MHA(Q,K,V)+S)

The next position-wise fully connected feedforward network also takes the form of residual connections, applied independently and identically to each position. This sublayer contains two linear transformations, activated in the middle by a Gaussian Error Linear Unit (GELU), and includes a dropout operation. The GELU activation function is formulated as follows:

(15) GELU(x)=xΦ(x)

where xΦ(x) is the cumulative distribution function of the standard Gaussian (normal) distribution and can be expressed as follows:

(16) $$\Phi (x)=\frac{1}{2}\left[1+erf(\frac{x}{\sqrt{2}})\right]$$

where *erf*(*x*) is the error function, which is a special function integral of the Gaussian distribution. Next, LN is performed. Finally, the sum of the input features and the output features is used as the result of the residual operation.

(17) E=LN(PE(O)+O)

where PF denotes the position-wise feed-forward operation.

### 2.4. Full Connectivity Layer

In the feature extraction module, the output features of the convolution module and the transformer encoder are fused to pass the extracted features from the CNN-LSTM directly to the classifier for processing and to perform the flattening operation. First, a dropout operation (dropout probability of 0.5) is applied to mitigate overfitting and improve the generalization of the model. These processed features are then fed into a fully connected layer containing *N* units, where *N* represents the number of categories in the MI EEG classification task. The loss function for the whole model uses cross-entropy, calculated as follows:

(18) $$L=-\frac{1}{M}\sum_{i}^{M}\sum_{j}^{N}y_{ij}\log({\bar{y}}_{ij})$$

where *M* is the number of samples in the EEG experiment, *N* is the number of categories, $y_{ij}$ is the true label of the jth category for the ith sample, and ${\bar{y}}_{ij}$ is the model’s probability of predicting the ith sample in the jth category.

### 2.5. Performance Indicators

In order to visually assess the classification performance of the model, this study uses several commonly used metrics for evaluation. Firstly, accuracy is introduced to indicate the proportion of correct predictions made by the model for all samples, which is calculated as follows:

(19) $$Accuracy=\frac{TP+TN}{TP+FN+FP+TN}$$

Second, the Kappa coefficient is introduced as a measure of classification performance, which integrates the stochastic consistency in the classification results, and is particularly applicable to the evaluation of the EEG motor imagery classification task, reflecting the reliability and consistency of the model in classifying motor imagery signals. Its calculation formula is as follows:

(20) $$k=\frac{P_{a}-P_{e}}{1-P_{e}}$$

The ROC (receiver operating characteristic) curve is also introduced to evaluate the classifier performance and compare the model’s performance in addressing this problem. The area under the ROC curve, i.e., the AUC value, quantifies the overall performance of the model. The closer its value is to 1, the better the performance of the model is. Two of the key metrics of the ROC curve, the true positive rate (TPR, true positive rate) and the false positive rate (FPR, false positive rate), are calculated as follows:

(21) $$TPR=\frac{TP}{TP+FN}$$

(22) $$FPR=\frac{FP}{FP+TN}$$

Among them, TP denotes the number of samples whose original data are positive and remain positive after classification; TN denotes the number of samples whose original data are negative and remain negative after classification; FN denotes the number of samples whose original data are positive but classified as negative; and FP denotes the number of samples whose original data are negative but are classified as positive. $P_{a}$ denotes the actual percentage of agreement, and $P_{e}$ denotes the expected percentage of agreement in a random situation.

In addition, the paired Wilcoxon Signed-Rank test was used to evaluate the statistically significant difference in the performance of the proposed model compared to the existing state-of-the-art methods. In the test results, if the *p*-value is greater than 0.05, it is considered that there is no statistically significant difference between the two methods; if the *p*-value is less than 0.05 and is marked with “*”, it indicates that the difference in performance of the two methods is statistically significant at the 95% confidence level; if the *p*-value is less than 0.01 and is marked with “**”, it indicates that the difference in performance reaches a high level of statistical significance at the 99% confidence level. This statistical approach provides us with an objective metric for determining whether model improvements have a significant impact.

### 2.6. Training Procedure

The model was trained and tested using the Pytorch 1.12.1 framework on a single NVIDIA GeForce RTX3050 Ti laptop GPU (NVIDIA Corporation, Santa Clara, CA, USA). The specific training configuration was as follows: the total number of training rounds was 1000, and an Adam optimizer with a learning rate of 0.001 and a batch size of 72 was used. At the same time, for specific subjects, a validation set ratio of 30% was adopted for real-time monitoring of the model’s performance. All hyperparameters were carefully tuned through multiple experiments to ensure that the model’s generalization ability and classification performance were optimized.

The LOSO (Leave-One-Subject-Out) method was utilized in the interdisciplinary evaluation. One of the nine subjects was selected as the test subject, one at a time, and the EEG data of the remaining eight were combined into a training set, and the process was repeated for each subject to ensure that the model could be trained on a diverse subset of data and complete the assessment. During model training, the learning rate, batch size, and number of training cycles were set to 0.001, 512, and 600, respectively. Meanwhile, the dropout ratio P1 was set to 0.25.

## 3. Experimental Results and Analysis

The experimental results confirm the remarkable effectiveness of the CLTNet model in the motor imagery EEG signal classification task. The model effectively captures the spatial, channel, time-series, and global properties of EEG signals through a preliminary process before deep feature extraction, thereby significantly improving the classification accuracy. This result provides a new methodology for improving brain–computer interface technology. Specifically, the CLTNet model showed excellent performance in tests on the BCI IV 2a and BCI IV 2b datasets, and the relevant results are detailed in Table 1.

In this study, the performance of the CLTNet model and its LOSO variant was systematically evaluated on the BCI Competition IV 2a and BCI Competition IV 2b datasets. The results of the analysis show that the CLTNet model has excellent performance and generalization ability when dealing with motion imagery (MI) BCI data, achieving an average accuracy of 83.02%, with a kappa coefficient of 0.77 on the BCI IV 2a dataset, while the average accuracy improved to 87.11%, with a kappa coefficient of 0.74 on the BCI IV 2b dataset. On the BCI IV 2a dataset, the average accuracy of the LOSO variant was 58.28%, with a kappa coefficient of 0.44, while on the BCI IV 2b dataset, the average accuracy of the LOSO variant was 76.26%, with a kappa coefficient of 0.53. These metrics indicate that the proposed EEG feature extraction model for motion images is effective and feasible in the four-class classification task.

**Figure 6:**
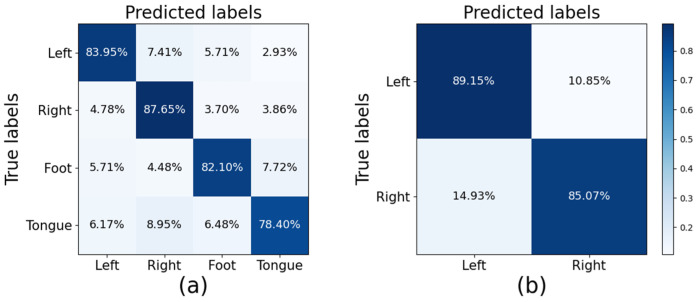
Average confusion matrices of the proposed CLTNet: (**a**) the BCI IV-2a dataset and (**b**) the BCI IV-2b dataset.

The average confusion matrix for the CTNet model is shown in Figure 6. Figure 6a shows the confusion matrix for the BCI IV-2a dataset, which shows that the left-hand movements were identified with the highest accuracy of 89.15%, while the tongue movements were the most difficult to identify, with an accuracy of only 78.40%. The most common misclassification was the misclassification of imagined tongue movements as imagined right-hand movements, with a misclassification rate of 8.95%. Figure 6b also shows the average confusion matrix for the BCI IV-2b dataset, where the decoding accuracies for left-hand and right-hand movements were 89.15% and 85.07%, respectively. The percentage of misclassifications in which the imagined left-hand movements were misidentified as right-hand movements was 10.85%, while the imagined right-hand movements were misclassified as left-hand movements 14.93% of the time.

## 4. Discussion

### 4.1. Ablation Experiment

The specific contribution of the different modules in the CLTNet model to classification performance was systematically evaluated by performing a series of ablation experiments. These experiments included removing the Transformer and LSTM modules, as well as the Spatial and S&R modules individually, and retaining only the pre-feature extraction network structure in combination with the single deep feature extraction module, in order to test the performance of the remaining network structure on the BCI IV-2a and IV-2b datasets. The effect of removing both the S&R and Transformer or LSTM modules was also investigated to understand the effect of the combined action of these modules. The experimental results are presented in Table 2.

In the BCI IV 2a dataset, removing the Transformer module reduced the accuracy of the model to 78.43%, with a Kappa coefficient of 0.71, while removing the LSTM module resulted in an accuracy of 79.86%, with a Kappa coefficient of 0.73. This suggests that although removing either module individually resulted in a decrease in performance, the LSTM module appears to have a greater impact on model performance than the Transformer module. When both the S&R and Transformer modules are removed, the accuracy drops significantly to 69.17%, with a Kappa coefficient of 0.59, demonstrating the importance of the S&R module in feature extraction. Further removal of the S&R and LSTM modules resulted in a slight decrease in accuracy to 77.62%, with a Kappa coefficient of 0.70, whereas the removal of the S&R module alone resulted in an accuracy of 71.80%, with a Kappa coefficient of 0.62. These results indicate that the S&R module is crucial for improving the classification performance of the model.

In the BCI IV 2b dataset, removing the Transformer module increased the accuracy to 86.14%, with a Kappa coefficient of 0.72, while removing the LSTM module further increased the accuracy to 86.90%, with a Kappa coefficient of 0.74. This is contrary to the results from the 2a dataset, suggesting that the dependence of the model on different modules may be different for different datasets. Removing both the S&R and Transformer modules reduced the accuracy to 81.85%, with a Kappa coefficient of 0.62, while removing the S&R and LSTM modules resulted in an accuracy of 85.84%, with a Kappa coefficient of 0.72. Removing the S&R module alone resulted in an accuracy of 82.38%, with a Kappa coefficient of 0.65. These results further confirm the critical role of the S&R module in improving model performance.

Overall, the CLTNet model showed high performance on both datasets, especially when all the feature extraction modules were included.

### 4.2. Experiments to Evaluate the Performance of the MHA

To evaluate the effect of the number of heads in the MHA module on the performance of the model, multi-head attention configurations of 2, 4, 8, and 16 heads were tested, where each head utilized query/key/value vectors of size 16, and their classification accuracies and Kappa values were recorded, as shown in Table 3.

The experimental results show that when the MHA module is set to two heads, the model performance is optimal, with a classification accuracy of 83.02% and a Kappa value of 0.77. As the number of heads increases to 4, 8, and 16, the classification performance gradually decreases, with accuracies of 79.51%, 79.20%, and 78.97%, respectively, and a decreasing trend in the Kappa value.

The MI-EEG dataset has a limited sample size, and as the number of heads increases, the model complexity increases significantly, making it difficult to achieve effective training and convergence on limited data. On the other hand, when the number of heads is small, the computational overhead and parameter size of the model are controlled, which is more suitable for optimization and generalization on small sample datasets. Choosing a lighter MSA module not only effectively improves classification performance, but also reduces the risk of overfitting, confirming the importance of appropriately adjusting module complexity when processing small-sample EEG data.

### 4.3. Comparative Experiments

In this study, the performance of the proposed ATCNet model is compared with the reproduced EEGNet [41], Shallow ConvNet [42], EEG Conformer [43], and CTNet [44] models using the BCI IV 2a and BCI IV 2b datasets. These models have some similarities with CLTNet in terms of structural design and are therefore comparable. The experimental results of the replicated models are based on the hyper-parameter settings defined in the original literature, while the data pre-processing, training, and evaluation processes follow the unified process defined in this study. The experimental results are shown in Table 4.

On the BCI IV 2a dataset, CLTNet achieved an accuracy of 83.02%, with a Kappa coefficient of 0.77, slightly higher than CTNet’s 79.86% and 0.73, but significantly higher than EEGNet’s 78.27% and 0.71, Shallow ConvNet’s 75.31% and 0.67, and EEG Conformer’s 73.34% and 0.64. In the LOSO evaluation, CLTNet’s accuracy remained at 58.28%, with a Kappa coefficient of 0.44, despite the degradation in performance of all models, demonstrating CLTNet’s strong generalization ability and adaptability to new subjects.

On the BCI IV 2b dataset, CLTNet performed particularly well, with an accuracy of 87.11% and a Kappa coefficient of 0.74, significantly surpassing all other models. This result fully demonstrates the superior performance of CLTNet when dealing with more complex motor imagery tasks. In particular, compared to EEGNet, Shallow ConvNet, and EEG Conformer, the accuracy of CLTNet is 0.94%, 2.32%, and 2.42% higher, and the Kappa coefficient is 0.02, 0.04, and 0.05 higher, respectively, showing the clear advantage of CLTNet in the classification task.

In the BCI IV 2a dataset, the accuracy and Kappa coefficient of the CLTNet model were significantly higher than those of the other models, with *p*-values lower than 0.05, specifically for EEGNet (*p* = 0.007), Shallow ConvNet (*p* = 0.003), EEG Conformer (*p* = 0.003), and CTNet (*p* = 0.039), indicating a statistically significant performance improvement for CLTNet. In the BCI IV 2b dataset, CLTNet’s accuracy and Kappa coefficient were also significantly higher compared to the other models, Shallow ConvNet (*p* = 0.007) and EEG Conformer (*p* = 0.027), further confirming CLTNet’s superior performance on this dataset. However, for EEGNet and CTNet, the *p*-values of 0.300 and 0.360, respectively, were greater than 0.05, indicating that the difference in performance between these models and CLTNet was not statistically significant. This suggests that although CLTNet outperformed EEGNet and CTNet on the BCI IV 2b dataset, this difference may not be entirely due to differences in the performance of the models themselves, but may be influenced by other factors.

**Figure 7:**
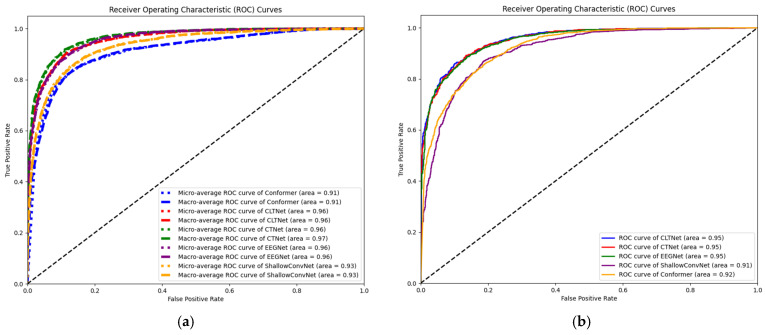
ROC curves for different models and their corresponding AUC values: (**a**) the BCI IV-2a dataset and (**b**) the BCI IV-2b dataset. (Conformer refers to the EEG Conformer model).

In this study, the ROC curves are further plotted and analyzed to provide insights into the performance of the CLTNet model on the BCI IV 2a and BCI IV 2b datasets, as well as its strengths and features compared to other models, as shown in Figure 7.

In the BCI IV 2a dataset, the micro-averaged and macro-averaged ROC curves of the CLTNet model both achieved an AUC of 0.96, a result that was second only to the best-performing CTNet (with an AUC of 0.97) and exceeded both EEGNet (with an AUC of 0.96) and Shallow ConvNet (with an AUC of 0.93). This demonstrates the efficient ability of CLTNet to discriminate different motor imagery tasks in the BCI IV 2a dataset with high classification accuracy and stability. In addition, the high AUC value of CLTNet highlights its excellent performance in resolving motor imagery EEG signals.

In the BCI IV-2b dataset, the AUC value of CLTNet’s ROC curve reaches 0.95, a performance that is on par with EEGNet and CTNet, and exceeds that of Conformer and Shallow ConvNet, which not only further validates the generalization ability and robustness of the CLTNet model across different datasets, but also, once again, demonstrates the excellent performance of CLTNet on both datasets. These results once again demonstrate that the hybrid deep learning model CLTNet has significant advantages in preliminary feature extraction, time-series data processing, and global feature modelling, which together contribute to the overall performance of the model.

### 4.4. Limitations and Future Work

Although the CLTNet model demonstrated excellent performance in subject-specific and cross-subject MI EEG decoding tasks on the BCI IV 2a and BCI IV 2b datasets, and its recognition accuracy exceeded that of several state-of-the-art methods, the model still has some limitations. Firstly, there is still room for improvement in the recognition accuracy of CLTNet in the MI-EEG decoding task for LOSO. Secondly, the S&R data enhancement strategy had no significant effect on the recognition accuracy of subject-specific MI-EEG decoding on the BCI IV 2b dataset.

To address these limitations, we plan to take the following steps in future work: Firstly, we will explore regularization strategies, specifically targeting cross-subject variability to improve the model’s recognition performance in cross-subject MI-EEG decoding. Finally, we will consider the use of Generative Adversarial Networks (GANs) to augment the training dataset, which may help to improve the model’s ability to generalize to new subjects and its robustness to noise. It is expected that this future work will further improve the performance of the CLTNet model, making it more effective and reliable in real-world BCI applications.

## 5. Conclusions

This study introduces CLTNet, a novel hybrid deep learning model for motor imagery classification designed for brain–computer interface (BCI) applications. Through comprehensive experiments on the BCI IV 2a and BCI IV 2b datasets, CLTNet demonstrated superior performance across multiple state-of-the-art methods, achieving an average accuracy of 83.02%, with a Kappa value of 0.77 on BCI IV 2a, and an average accuracy of 87.11%, with a Kappa value of 0.74 on BCI IV 2b. These results demonstrate the effectiveness of CLTNet in feature extraction and decoding of EEG signals associated with motor imagery tasks.

The innovative fusion of CNN, LSTM, and Transformer modules in CLTNet allows for the comprehensive extraction of local features, temporal dynamics, and global dependencies in EEG signals. This multidimensional feature extraction capability is essential for improving the accuracy and robustness of the model in classifying MI-EEG signals.

## Figures and Tables

**Table 1:** The classification accuracy percentages and Kappa values on the BCIC IV 2a and BCIC IV 2b datasets using CLTNet.

Subject	BCI IV 2a				BCI IV 2b			
	CLTNet		CLTNet(LOSO)		CLTNet		CLTNet(LOSO)	
	%	*k*	%	*k*	%	*k*	%	*k*
A01T	88.54	0.85	71.35	0.62	75.94	0.52	77.08	0.54
A02T	68.06	0.57	38.72	0.18	69.29	0.39	67.94	0.36
A03T	95.49	0.94	83.33	0.78	84.68	0.69	65.56	0.31
A04T	81.60	0.75	53.13	0.38	97.81	0.96	81.21	0.62
A05T	80.56	0.74	43.75	0.25	97.50	0.95	84.32	0.69
A06T	68.40	0.58	35.07	0.14	85.31	0.71	75.42	0.51
A07T	90.63	0.88	70.31	0.60	93.13	0.86	81.53	0.63
A08T	87.85	0.84	67.19	0.56	91.88	0.84	75.00	0.50
A09T	86.11	0.81	61.63	0.49	88.44	0.77	78.33	0.57
Mean	83.02	0.77	58.28	0.44	87.11	0.74	76.26	0.53

**Table 2:** Results of the ablation study based on different approaches of the preliminary feature extraction module on the BCIC IV 2a and BCIC IV 2a datasets, where w/o indicates that there is no corresponding block.

Dataset	Method	Accuracy %	Kappa
BCIC IV 2a	w/o Transformer	78.43	0.71
	w/o LSTM	79.86	0.73
	w/o S&R, Transformer	69.17	0.59
	w/o S&R, LSTM	77.62	0.70
	w/o S&R	71.80	0.62
	CLTNet	83.67	0.77
BCIC IV 2b	w/o Transformer	86.14	0.72
	w/o LSTM	86.90	0.74
	w/o S&R, Transformer	81.85	0.62
	w/o S&R, LSTM	85.84	0.72
	w/o S&R	82.38	0.65
	CLTNet	87.11	0.74

**Table 3:** Multi-head attention experiment based on BCI IV-2a dataset.

Heads	Accuracy %	Kappa
2	83.02	0.77
4	79.51	0.73
8	79.20	0.72
16	78.97	0.71

**Table 4:** Classification performance of different models on BCI IV 2a and BCI IV 2b datasets in terms of accuracy %, Kappa coefficient, and *p*-value (* *p* < 0.05, indicating that the performance difference between the two methods is statistically significant at the 95% confidence level. ** *p* < 0.01, indicating that the performance difference between the two methods reaches a high level of statistical significance at the 99% confidence level).

Dataset	Method	Accuracy %	Kappa	*p*-Value
BCIC IV 2a	CLTNet	83.02	0.77	-
	EEGNet	78.27	0.71	0.007 **
	Shallow ConvNet	75.31	0.67	0.003 **
	EEG Conformer	73.34	0.64	0.003 **
	CTNet	79.86	0.73	0.039 *
	CLTNet (LOSO)	58.28	0.44	**-**
	EEGNet (LOSO)	58.11	0.45	-
	Shallow ConvNet (LOSO)	56.46	0.42	-
	EEG Conformer (LOSO)	54.76	0.40	-
	CTNet (LOSO)	58.15	0.43	-
BCIC IV 2b	CLTNet	87.11	0.74	-
	EEGNet	86.17	0.72	0.300
	Shallow ConvNet	84.79	0.70	0.007 **
	EEG Conformer	84.69	0.69	0.027 *
	CTNet	86.88	0.73	0.360
	CLTNet (LOSO)	76.27	0.53	-
	EEGNet (LOSO)	77.22	0.54	-
	Shallow ConvNet (LOSO)	76.06	0.57	-
	EEG Conformer (LOSO)	72.89	0.46	-
	CTNet (LOSO)	77.29	0.55	-

## Data Availability

Publicly available datasets were used in this study. These data can be found here: https://www.bbci.de/competition/download/competition_iv/BCICIV_2a_gdf.zip (accessed on 15 January 2025). https://www.bbci.de/competition/download/competition_iv/BCICIV_2b_gdf.zip (accessed on 15 January 2025).
